# Supplementary figures and images for: Identification and Validation of Candidate Genes Conferring Resistance to Downy Mildew in Maize (Zea mays L.)
Source: Genes (Basel). 2020 Feb 11;11(2):191. doi: 10.3390/genes11020191 (PMC7074223; doi:10.3390/genes11020191)

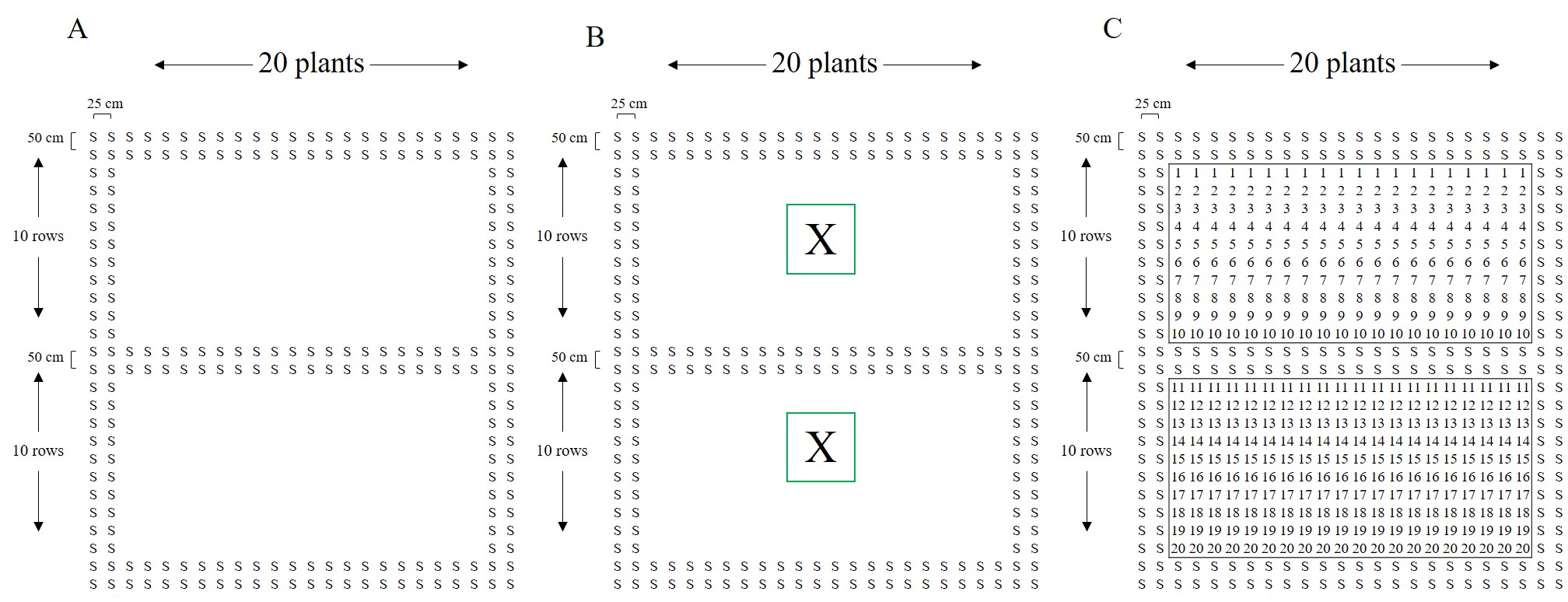

Supplement: Supplementary file 1 [file genes-11-00191-s001.zip › Figure S1.jpg]

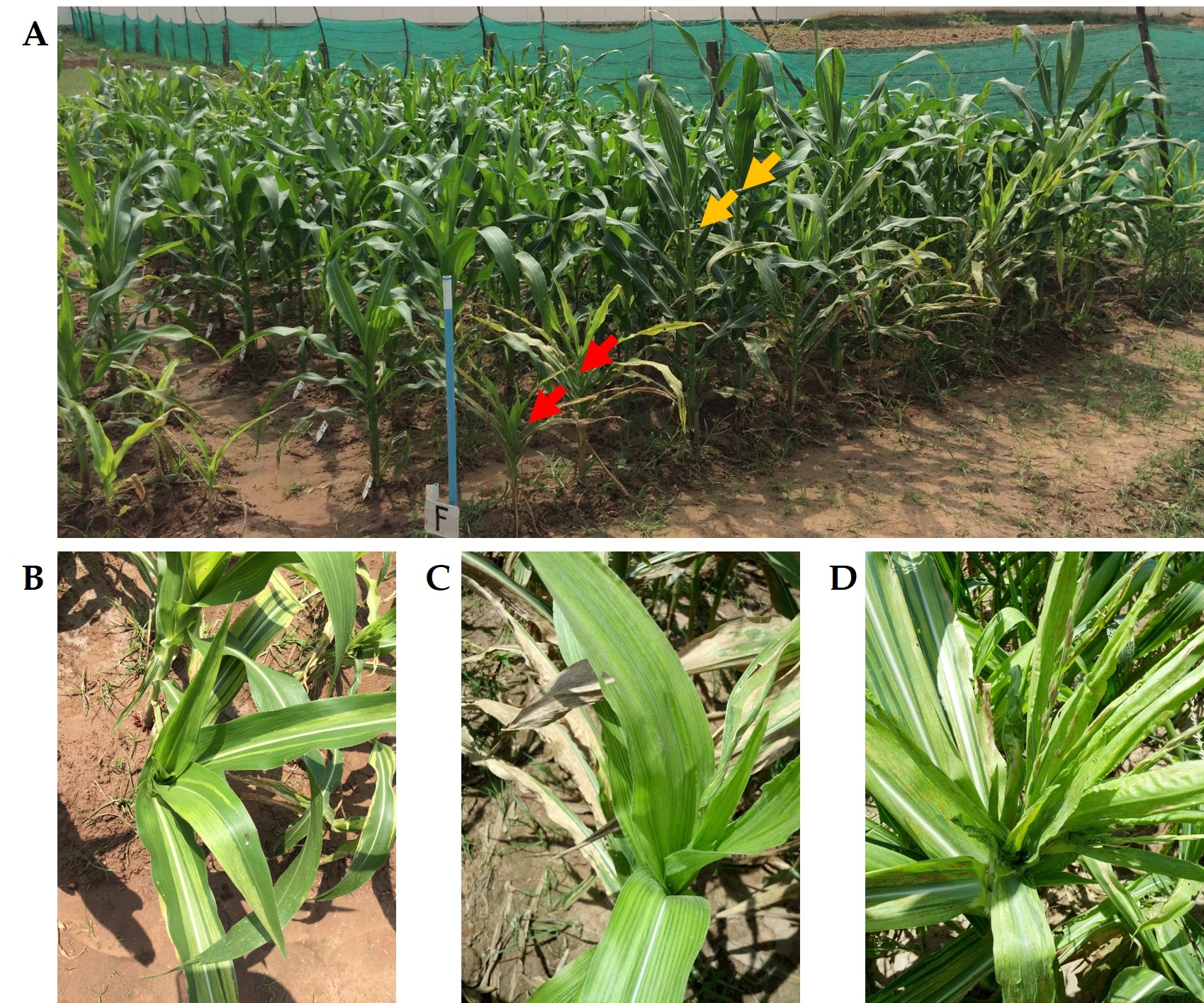

Supplement: Supplementary file 1 [file genes-11-00191-s001.zip › Figure S2.jpg]

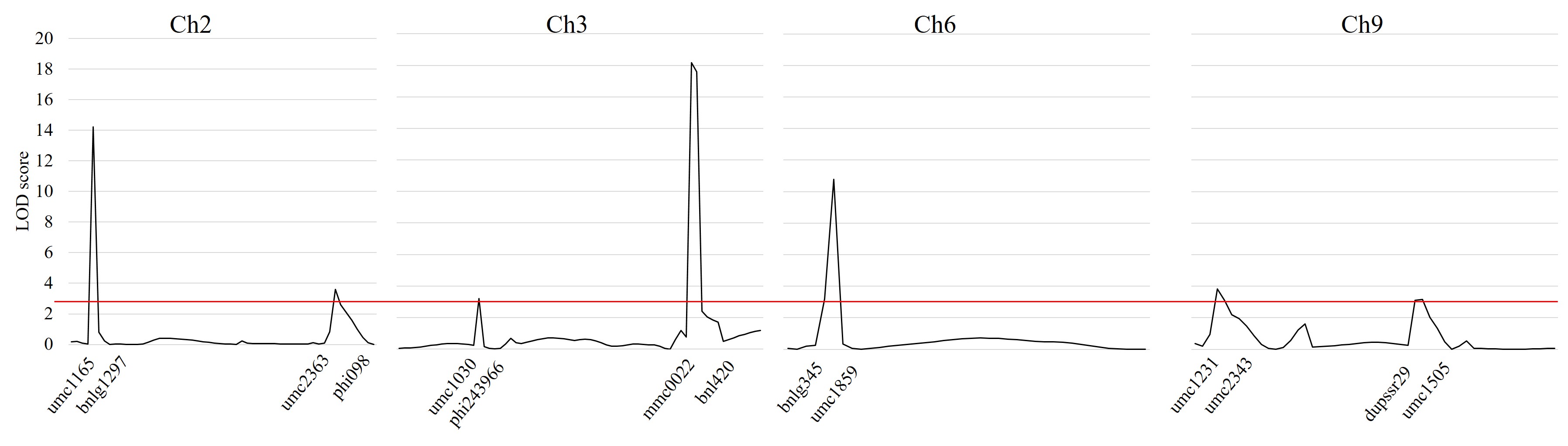

Supplement: Supplementary file 1 [file genes-11-00191-s001.zip › Figure S3.jpg]

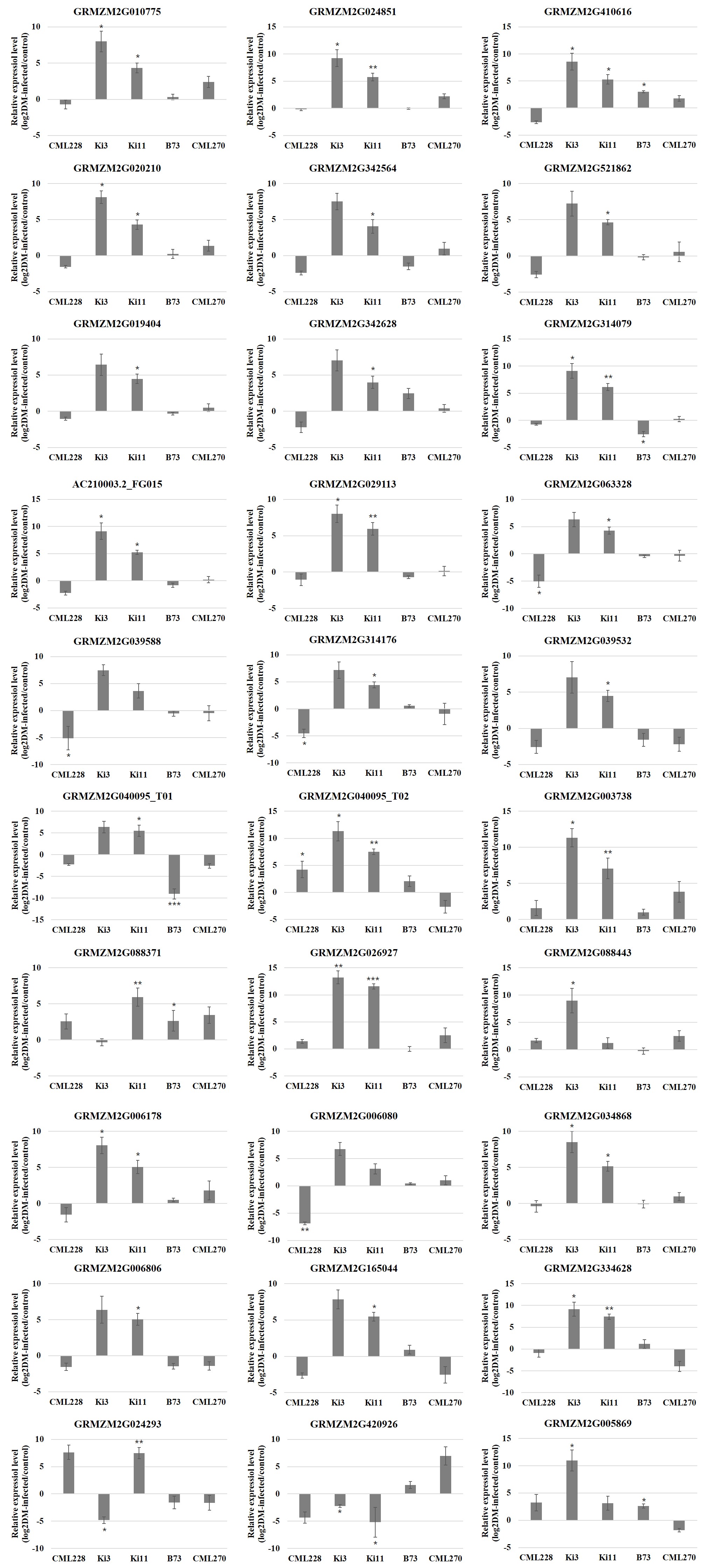

Supplement: Supplementary file 1 [file genes-11-00191-s001.zip › Figure S4.jpg]
